# Pediatric Obesity and the Immune System

**DOI:** 10.3389/fped.2019.00487

**Published:** 2019-11-22

**Authors:** Giuseppina Rosaria Umano, Carmelo Pistone, Enrico Tondina, Alice Moiraghi, Daria Lauretta, Emanuele Miraglia del Giudice, Ilaria Brambilla

**Affiliations:** ^1^Department of the Woman, Child, General and Specialized Surgery, University of Campania Luigi Vanvitelli, Naples, Italy; ^2^School of Pediatrics, University of Pavia, Pavia, Italy; ^3^Department of Pediatrics, Fondazione IRCCS Policlinico San Matteo, University of Pavia, Pavia, Italy

**Keywords:** obesity, immune system, inflammation, adipokines, atopy, autoimmune diseases

## Abstract

Obesity has reached pandemic proportion and represents a major risk for several comorbidities. In addition to metabolic and cardiovascular obesity-related diseases, recent evidence suggested that obesity might affect immune system function. Adipose tissue is considered an endocrine organ that actively secretes cytokines also referred to as “adipokines.” Adipokines play an important role in the control of human metabolism. The dysfunctional adipose tissue in obese individuals is characterized by an altered cytokine secretion pattern that promotes chronic low-grade inflammation. Epidemiological evidence highlights the association between obesity and allergic and immune-mediated diseases, such as asthma, allergic rhinitis, rheumatic arthritis, and psoriasis. Less is known about underlying pathogenic mechanisms. However, several recent *in vivo* and *in vitro* studies have reported that adipokines are involved in inflammatory and autoimmune disorders by influencing both innate and acquired immune responses. In addition, obesity has been associated with reduced immune surveillance and increased risk of cancer. This paper reviews the evidence regarding the role of adipokines in immune system regulation, with particular emphasis on autoimmune, allergic, and inflammatory disorders. Understanding how obesity affects immune system functions may enable researchers to find new potential therapeutic targets in the management of allergic and autoimmune diseases.

## Introduction

The obesity epidemic represents a major health issue of the twenty-first century. The prevalence of the disease has significantly increased in the last decades worldwide, including low-income countries (1). In addition, it has been reported that pediatric obesity significantly increases the risk of obesity and its cardiovascular complications during childhood and adulthood (2, 3).

The constant increase in obesity prevalence might be ascribed to changes in lifestyle habits, namely, assumption of junk food and sedentary behavior. Moreover, genetic background plays a role by affecting energy expenditure and food intake (4). The excess of fat present in obese individuals results in a hypertrophic white adipose tissue with a consequent derangement in adipocytes metabolic activity. Adipose tissue in some obese subjects displays a pro-inflammatory activity and has been associated with obesity-related comorbidities in both adults and children (5–8). Adipocytes actively secrete several hormones and cytokines, also referred to as adipokines that exert metabolic and immunological functions, as they could modulate the innate and acquired immune cell activity. Moreover, they are capable of promoting pro-inflammatory signals leading to a chronic systemic low-grade inflammation. In addition, growing evidence reports the association between obesity and immune disorders, such as cancer, autoimmunity, and atopy (9). Therefore, the altered secretion pattern of adipokines in obesity is considered the link between obesity and its metabolic and immunological comorbidities (10).

In this review, we summarize the current knowledge on the basis of endocrine and immunological functions of white adipose tissue. In particular, we describe the interplay between adipokines and both innate and acquired immunity. Moreover, we report the evidences and possible mechanisms of the association between pediatric obesity and immunological disorders.

## Obesity and Inflammation

Clinical studies have observed a significant association between serum markers of chronic low-grade inflammation and obesity-related comorbidities in children and adolescents (11–13). Serum levels of C-reactive protein (CRP) directly correlate with insulin resistance and carotid intima-media thickness (cIMT) in children and adolescents with overweight and obesity (11). Moreover, it has been reported that white blood cell count is predictive of increased cIMT and ventricular hypertrophy in obese children and adolescents (12). In addition, weight loss after lifestyle intervention trial was effective in reducing both serum inflammatory markers and insulin resistance in obese children and adolescents (14).

Inflammation is a mechanism that involves a series of non-cellular and cellular mediators involved in the response to infections, tissue damage, cellular death, and cancer. Obesity is accompanied by a sterile chronic low-grade inflammation in the adipose tissue that is not related to infection or tissue damage (15). This process is also referred to as metabolic inflammation or metainflammation. Metainflammation is an active player in the development of obesity-related comorbidities, as obesity-related inflammation might impair the function of other organs (16). Adipose tissue immune response is mediated by adipose tissue-resident immune cells, namely, macrophages, mast cells, neutrophils, and T and B lymphocytes, which are the second more represented cellular type, after adipocytes (17). Mechanisms triggering adipose tissue inflammation are not completely clear. However, adipose tissue hyperplasia and hypertrophy occurring in obese individuals may exert an important role (18). In fact, an increase of adipocyte size leads to hypoxia, intracellular oxidative stress, and release of pro-inflammatory molecules (18). However, some studies have shown that metabolically unhealthy obese adolescents tend to have smaller adipose cells (19), which allows for a smaller storage capacity and favor a more pronounced flux of free fatty acids. Inflammasome activation in subcutaneous adipose tissue (SAT) has been associated with an altered abdominal fat distribution (19). The inflammasome chronic activation impairs the SAT storage capability leading to a lipid spill over into visceral adipose tissue (VAT) (19).

The excess of free fatty acids contributes to an unfavorable metabolic phenotype probably through the macrophage activation in adipose tissue. In fact, free fatty acids directly activate immune cells and induce secretion of pro-inflammatory mediators through interacting with toll-like receptors (TLRs) (20). In VAT, adipocytes develop a dysfunctional phenotype characterized by smaller adipose cells and expression of adhesion molecules for monocytes/macrophages (21).

### Macrophages

Macrophages are the most common immune cell type in adipose tissue. The number of macrophages has been associated with size of adipocytes and severity of obesity (21). A small amount of adipose tissue macrophages (ATMs) derives from pre-adipocyte maturation, while the majority comes from systemic circulation (22). Hypertrophic adipose tissue promotes macrophage migration and infiltration via secretion of chemokines. Several chemokines are involved in this pathway. Among them, monocyte-chemoattracting protein 1/chemokine C–C motif receptor 2 (MCP1/CCR2) binds macrophage receptor CCR2, leading to infiltration (23). Moreover, obesity alters the phenotype and distribution of macrophages. Macrophages could be distinguished in two subsets according to membrane antigen and cytokine secretion: classically activated M1 type and alternative activated M2 type. M1 type exerts a pro-inflammatory and antibacterial role; it expresses receptor for inflammatory mediators, such as granulocyte-macrophage colony-stimulating factor (GM-CSF), IFN-γ, and lipopolysaccharides (LPS). Conversely, M2 phenotype is induced by anti-inflammatory molecules, namely, IL-13, IL-10, IL-4, and macrophage colony-stimulating factor (M-CSF), and displays anti-inflammatory and anti-parasitic functions (24, 25). The different polarization of macrophages in human adipose tissue is influenced by nutritional status. In normal weight subjects, the prevalence of eosinophils, T-lymphocytes, and natural killer (NK) cells promote a polarization toward M2 type. These macrophages, in a favorable environment, exert homeostatic actions, regulating cellular proliferation, extracellular matrix deposition, and removing cellular debris in stromal matrix. In obese subjects, oxidative stress, necrotic cellular debris, and overload of free fatty acids lead to a polarization toward an M1 phenotype. In contrast to M2, M1 macrophages do not migrate in stromal matrix; they tend to create aggregates surrounding necrotic adipocytes, in a typical crown-like structure (26). In humans, the number of M1 macrophages directly correlates with systemic inflammation, insulin resistance, type 2 diabetes, and fatty liver disease (15).

### Lymphocytes

The second largest immune cell subset in adipose tissue is represented by T-lymphocytes. T-cells could be divided in CD4+ and CD8+ according to surface antigens. The cytokine secretion pattern of CD4+ allows a further categorization in Th1 (INF-γ), Th2 (IL-4, IL-5, and IL-13), Treg (IL-10 and transforming growth factor B), and Th17 (IL-17, IL-21, and IL-22). CD8+ are cytotoxic cells that secrete perforins, granzymes, and a variety of cytokines that mediate activation of other immune cells. In dysfunctional adipose tissue, the T-lymphocyte subpopulation pattern is polarized toward a pro-inflammatory activity. Therefore, Th1, Th17, and CD8+ are more represented compared to anti-inflammatory Th2 and Treg (15).

### Other Adipose Tissue-Resident Immune Cells

Neutrophils, mast cells, and dendritic cells constitute a small fraction of immune cells in adipose tissue. Nevertheless, they play an important role in amplifying inflammation process via secretion of pro-inflammatory mediators. The amount of eosinophils, instead, is reduced. They promote M2 macrophage and Th2 differentiation and suppress inflammatory stimuli (15).

## Adipokines as Immune Modulators

**Figure 1:**
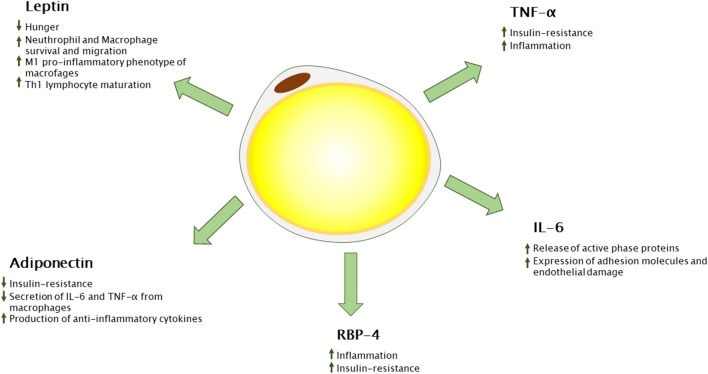
Adipokines secreted from white adipose tissue and their immunological functions. RBP-4, retinol binding protein 4; TNF-α, tumor necrosis factor alpha; Th-1, T-helper 1.

In the last years, a number of adipose tissue-derived cytokines have been characterized, the so-called adipokines (Figure 1). Besides adipocytes, the vascular-stromal component of adipose tissue concurs with cytokine secretion in an adiposity-dependent manner. Adipokines play a crucial role not only in energy homeostasis but also in inflammatory and immune reaction, most of them promoting inflammation (27).

### Leptin

**Figure 2:**
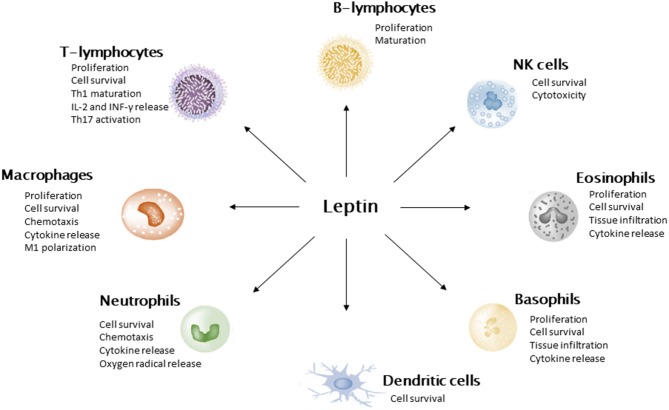
Leptin action on immune cells. Leptin promotes cell survival, stimulates the secretion of pro-inflammatory cytokines, and suppresses cell apoptosis. It induces M1 polarization of macrophages and stimulates Th1 pathway in T-lymphocytes.

Leptin was first described by Zhang et al. (28) in a murine model. It is encoded by murine *ob* gene and human homologous *LEP* gene. Animals and human models showed that inhibition of leptin action results in food assumption and insulin resistance (27, 28). Leptin secretion is positively correlated with adipose tissue mass; therefore, obese subjects show high leptin plasmatic levels. Nevertheless, obese subjects develop leptin resistance with a decreased sensitivity to anorexinergic hormone stimulus. Leptin has pleiotropic activities. The main role is suppression of food assumption via inhibiting hypothalamic nuclei that stimulate hunger and stimulation of those which promote satiety (29). Leptin loss of function is associated with hyperphagia, rapid weight gain, and insulin resistance (30). Moreover, leptin influences pubertal development and shows immunomodulatory activity (31). In fact, several immune cell types express leptin receptor (LEPR) (Figure 2). In neutrophils, leptin activates anti-apoptotic signals, resulting in cell survival. In addition, it induces neutrophil activation in terms of chemotaxis, tissue infiltration, and oxygen radicals' release. Similarly, eosinophils and basophils express LEPR on the cellular surface. As for neutrophils, leptin acts as a survival cytokine for these cells and stimulates tissue infiltration and pro-inflammatory molecule release (32). Moreover, leptin is crucial for monocyte/macrophage function. In experimental models of *LepR*, knock-out mice impaired macrophages killing activity and phagocytosis has been reported (33). Additionally, leptin has been shown to enhance monocyte and macrophage survival, migration, release of pro-inflammatory cytokines (namely, IL-6 and TNF-α), and expression of surface markers (CD39, CD69, CD25, CD71, and IL-1Rα). Leptin also stimulates proliferation and cytotoxicity of NK cell and constitutes a survival cytokine for dendritic cells. With regard to adaptive immune response, hormone inhibits T-lymphocyte apoptosis, promotes Th1 response, suppresses Th2 activity, and enhances IL-2 and INF-γ production. Moreover, it sustains Th17 pro-inflammatory function and B-lymphocyte activity (34). In general, leptin displays a pro-inflammatory activity, interacting with both innate and adaptive immune system. These data highlight the crucial role of leptin in pathogenesis of obesity-related comorbidities, in terms of metabolic derangement and chronic inflammation.

### Resistin

Resistin is mainly secreted by monocytes and macrophages and a small fraction derives from adipocytes. It is associated with insulin resistance as it inhibits insulin receptor signaling via suppression of cytokine signaling-3 (SOCS-3). Consequently, it can influence plasmatic glucose levels and insulin sensitivity (35). Moreover, it stimulates the release of IL-6 and TNF-α from neutrophils and counteracts adiponectin anti-inflammatory activity on endothelial cells (36).

### Interleukin-6 (IL-6)

One of the most important pro-inflammatory cytokines is IL-6. About one-third of total circulating levels of IL-6 derives from adipocytes (37). IL-6 directly stimulates secretion of active phase proteins, such as CRP and fibrinogen. Waist circumference mainly correlates with IL-6 and CRP plasma levels, highlighting the crucial role of visceral adiposity in inflammation (38). In addition, it promotes expression of adhesion molecules on endothelial cells for leucocytes, enhancing vascular damage and inflammation (38, 39).

### Tumor Necrosis Factor-Alpha (TNF-α)

TNF-α has a central role in pathogenesis of several inflammatory and autoimmune diseases. Monocyte/macrophage cells are responsible for the major fraction of serum TNF-α. In addition, cytokine levels positively correlate with adiposity measures and insulin resistance. TNF-α inhibits the expression of PPAR-γ (peroxisome proliferator-activated receptor-gamma). PPAR-γ exerts anti-inflammatory activity throughout, reducing pro-inflammatory cytokine secretion in human macrophages (40). Furthermore, it interferes with insulin receptor IRS-1 phosphorylation, leading to insulin resistance (41). Moreover, TNF-α antagonist treatment in subjects affected by autoimmune diseases improves insulin sensitivity (42). However, these findings have not been confirmed in models of obesity-related insulin resistance (43).

### Retinol Binding Protein-4 (RBP-4)

Retinol-binding protein 4 (RBP-4) is an adipokine involved in the pathogenesis of insulin resistance. In particular, it reduces insulin sensitivity, decreasing IRS-1 insulin-induced phosphorylation. Hepatocytes are the main source of RBP-4, while visceral adipocytes and macrophages secrete a minor fraction. However, RBP-4 is secreted in a dependent manner to VAT mass, as it has been suggested as a marker of visceral adiposity and chronic low-grade inflammation (43).

**Figure 3:**
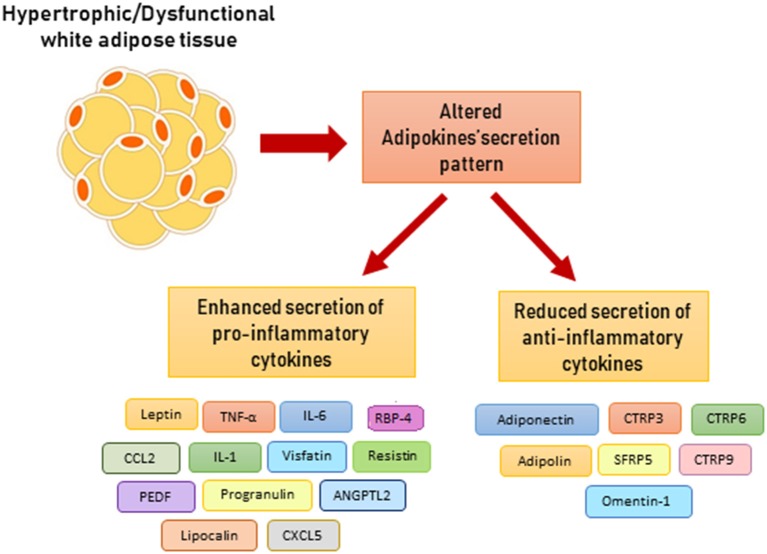
Altered adipokine secretion pattern in dysfunctional adipose tissue. ANGPTL2, angiopoietin-like protein 2; CCL2, CC-chemokine ligand 2; CXCL5, CXC-chemokine ligand 5; CTRP, C1q/TNF-related protein-3; CTRP, C1q/TNF-related protein-6; CTRP, C1q/TNF-related protein-9; PEDF, pigment epithelium-derived factor; RBP-4, retinol binding protein 4; SFRP5, secreted frizzled related protein 5.

A variety of pro-inflammatory adipokines have been described, namely, visfatin, lipocalin, angiopoietin-like protein 2 (ANGPTL2), CC-chemokine ligand 2 (CCL2), and CXC-motif chemokine ligand 5 (CXCL5) (Figure 3). However, their role in metainflammation has not been completely understood (43).

### Adiponectin

Adipose tissue releases a small amount of adipokines with an anti-inflammatory activity, among them adiponectin is the best characterized (Figure 3). Adiponectin stimulates fatty acid oxidation and glucose uptake in skeletal muscle and liver, thus improving insulin sensitivity (44). In macrophages, adiponectin signal promotes an M2 phenotype polarization, reduction of TNF-α secretion, and enhancement of scavenger activity. Moreover, it stimulates release of anti-inflammatory IL-10. Pro-inflammatory cytokines (IL-6 and TNF-α), oxidative stress, and adipocyte hypoxia decrease adiponectin secretion. In fact, clinical studies have reported that hormone levels are inversely correlated with CRP plasma levels (45). Moreover, obesity is associated with lower adiponectin levels and increased risk of cardiovascular diseases (46, 47). Similarly to adiponectin, the C1q/TNF-related proteins (CTRPs) promote anti-inflammatory pathway. Some CTRPs are expressed in adipose tissue, such as CTRP3, CTRP6, CTRP9, and CTRP12, and inhibit macrophage pro-inflammatory activity (48). In addition, omentin-1 is secreted from VAT and reduces vascular inflammation *via* inhibition of endothelial adhesion molecules expression (48).

In conclusion, adipokine secretion pattern from adipose tissue exerts a central role in the link between obesity and altered immune response, leading to systemic inflammation and reduced immune tolerance.

## Obesity, Atopy, and Asthma

**Figure 4:**
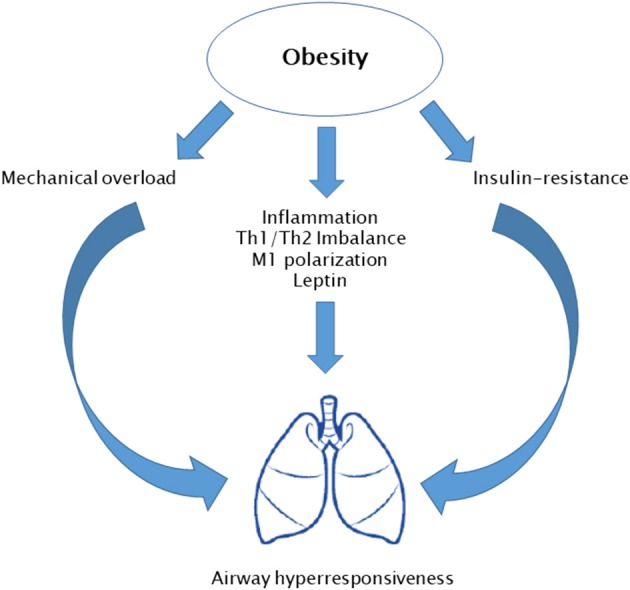
Potential mechanisms involved in obesity-related asthma.

Paralleling with the obesity epidemic, a rise in the prevalence of allergic diseases has been registered. The higher exposure to indoor and outdoor air pollutants has a crucial role in this phenomenon. However, evidence suggests that obesity and obesity-related inflammation might be at least in part responsible of this process. Data from the National Health and Nutrition Examination Study III (NHANES III) have reported a positive association between BMI and atopy rates (49). Nevertheless, obese subjects do not show a significant increase of serum atopy markers, such as IgE plasma levels and eosinophils count (50, 51). In addition, evidence about a possible link between obesity and other allergic disorders, namely, allergic rhinitis and atopic dermatitis, is sparse. Therefore, this field needs to be more deeply investigated. Conversely, several data are available about the relation between asthma and obesity in both adults and children (9). Cross-sectional epidemiological studies have reported that obese children are frequently affected by asthma (49, 52, 53). Moreover, a metanalysis including six perspective studies found that obese children show a twofold higher risk for asthma compared to normal weight children (54). In addition, obese children commonly show a more severe asthma phenotype, which tend to be resistant to pharmacologic therapies with frequent exacerbations. Nevertheless, obese children do not show elevation of expiratory levels of inflammatory markers, such as exhaled nitric oxygen (eNO). Several underlying mechanisms have been proposed for obesity-related asthma (Figure 4). In particular, excess of truncal adiposity leads to a mechanical overload to respiratory muscles. This results in reduced functional residual capacity, decreased residual volume, and expiratory reserve volume. This volume reduction exposes obese children to an impairment of forced expiratory volume in 1 s and forced vital capacity ratio (FEV1/FVC). In addition to mechanical overload, metabolic derangement might also play a role in obesity-related asthma. Dyslipidemia and insulin resistance have been associated with an impaired FEV1/FVC ratio. Insulin is a trophic stimulus for low airway smooth muscle cells. It stimulates laminin production through phospho-inositide-3 kinase/Akt (PI3K/AKT) pathway, leading to muscle hypertrophy. Additionally, it enhances airway hyper-responsiveness via stimulating parasympathetic innervation. These mechanisms promote airway obstruction during physical exercise and a perception of respiratory effort during inspiration (55). Moreover, chronic low-grade inflammation occurring in obesity plays an important role. In obese children, imbalance between Th1/Th2 phenotype toward a Th1 reaction has been related to decrease FEV1/FVC ratio. Similarly, macrophage pro-inflammatory M1 type induces airway obstruction (56). Finally, derangement of adipokines' milieu has a role in adult asthma. In particular, leptin serum levels are inversely correlated with pulmonary volumes and FEV1/FVC ratio, while it increases bronchial hyper-responsiveness (57). However, most of the available evidence is based on cross-sectional studies, and this makes it difficult to completely understand underlying mechanisms of obesity-related asthma.

## Obesity and Autoimmunity

The characterization of immunological function of adipose tissue has suggested a possible link between obesity and autoimmune disorders. Autoimmunity is strongly influenced by genetic background; however, environmental factors are central in the beginning of immune response. Several observational studies have reported the association between obesity and a number of autoimmune diseases, namely, autoimmune thyroiditis, type 1 diabetes, multiple sclerosis, rheumatic arthritis, systemic lupus erythematosus, inflammatory bowel diseases, and psoriasis. However, data for pediatric age are sparse. Longitudinal studies have reported that obese children and adolescents display a twofold higher risk of developing multiple sclerosis in adulthood; this risk is higher in girls compared to boys and increases in subjects with a predisposing genetic background (HLA DRB1^*^15) (58–60). Several potential underlying mechanisms have been proposed: vitamin D deficiency, imbalance of macrophages M1/M2 phenotypes, increased levels of leptin, and reduced adiponectin levels (60). With regard to rheumatoid arthritis (RA), obese subjects present a 20% higher risk of RA in longitudinal studies (61). In addition, RA severity and treatment responsiveness is negatively influenced by adiposity, as obese subjects are more prone to develop a severe disease with reduced remission and high rate of comorbidities (62). Adipokines are involved in joint damage; they stimulate secretion of pro-inflammatory cytokines and metalloproteinases from synovial chondrocytes and fibroblasts (63). Conversely, no solid evidence is available to confirm the hypothesized relationship between obesity and systemic lupus erythematosus pathogenesis, while it has been reported that obesity increases disease activity (64, 65). More solid evidence confirms that obesity and psoriasis are correlated (66), but the pathogenic underpinnings of this association are still debated. Subjects with psoriasis and psoriatic arthritis tend to gain weight because of a sedentary lifestyle; obesity worsens disease severity and the risk of comorbidities (67). Additionally, weight loss improves disease control and response to treatment (68). Several mechanisms underlie this observation. First of all, altered adipokines' milieu characterized by a prevalence of IL-6, TNF-α, and leptin promotes immune cell activation, proliferation, and migration (30). Moreover, reduction of Treg cells with a concurrent increase of Th17 has been associated with autoimmunity (11). Nutrients also play a role, as a western diet with high-fat and high-sugar content leads to intestinal dysbiosis and Th17/Treg imbalance (69).

Furthermore, adipose tissue is involved in peripheral aromatization of androgens to estrogens, and this may represent a means through which excess adiposity might predispose to autoimmunity. In fact, females are more affected by autoimmune diseases compared to males. This observation might be related to the influence of sexual hormones on immune tolerance. Interestingly, women show higher leptin plasma levels compared to men. It is well-known that estrogens and dehydroepiandrosterone (DHEA) are the major mediators of females' susceptibility to autoimmune disorders. These hormones promote immunoglobulin release, stimulate adaptive immune responses, and induce secretion of pro-inflammatory cytokines (70).

As for RA, adipokines are central players in this scenario. Leptin and resistin plasma levels are elevated in subjects with psoriasis. Moreover, *in vitro* experiment showed that leptin stimulates cytokine release in human keratinocytes. Contrasting results have been produced about the role of adiponectin in psoriasis pathogenesis and severity (63). In conclusion, more studies are needed to confirm and investigate the pathophysiological mechanisms underlying the potential relationship between autoimmunity and obesity.

## Obesity and Cancer

Previous studies have shown that nutritional status affects immunocompetence, as both undernutrition and overweight influence immune system functions. Epidemiological studies reported that subjects with obesity are at higher risk of infectious disease, infectious disease-related complications, and cancer (71, 72). In regard to cancer, it has been estimated that up to 50% of a variety of cancer types, namely, endometrial, breast, colon, liver, and prostate, might be attributed to obesity in adults (73). Additionally, a growing body of evidence suggests that pediatric obesity might increase the risk for cancer occurrence in adulthood. As for atopy and autoimmunity, more complex mechanisms interplay in obesity-related carcinogenesis. The chronic low-grade systemic inflammation has been recognized as the trigger of carcinogenesis and cytokines exert a crucial role in this process. IL-6 and IL-1β promote cell proliferation and survival. Moreover, TNF-α can increase DNA damage and cell proliferation through the NF-KB pathway with upregulation of anti-apoptotic proteins (74). In addition, IL-6 induces transcription of genes involved in angiogenesis, invasiveness, and metastasis via STAT3 activation. Similarly, leptin activates STAT3 pathway, enhancing cell proliferation and angiogenesis. Conversely, adiponectin has an anti-inflammatory and anti-tumorigenic activity through activation of AMPK (adenosine monophosphate-activated protein kinase) and inhibition of mTORC1 and other tumorigenic mediators. Therefore, dysfunctional hypertrophic adipose tissue secretes a relatively higher amount of carcinogenic mediators over anti-tumorigenic molecules. On the other hand, obesity is associated with impaired immune surveillance as suggested by increased risk of infection and lower response to immunization. Obese subjects have a reduced number of NK, dendritic, and CD8+ cells that mediate cytotoxic functions (75). In addition, Michelet et al. observed that NK cells from obese humans secrete lower amounts of INF-γ. The authors hypothesized that free fatty acid overload induces a shift in NK cell metabolism from glycolysis to lipid beta-oxidation, thus deranging cellular homeostasis and function. The direct consequence of reduced immune surveillance is tumor growth and invasiveness (76).

To date, data in pediatric age are sparse, epidemiological evidence comes from adult population, and potential mechanisms have been investigated only in mice. However, it might be hypothesized that these processes might initiate already during childhood.

## Conclusions

Obesity is a major risk factor for allergic, autoimmune, and neoplastic diseases, and it is at least in part responsible for the recent burden of these disorders. Dysfunctional adipose tissue is the key pathogenic factor leading to this association. However, to date, a small number of studies have addressed this issue in obese children and adolescents; therefore, more studies are needed to confirm these observations and to understand the complex interplay between adipose tissue and immune system. The gain in knowledge in metainflammation patterns and mediators might allow the development of novel treatments for pediatric obesity. To date, there are no studies evaluating inflammatory targets in the treatment of pediatric obesity. However, PPAR-γ and PPAR-α agonists have shown to modulate monocyte function in activated human adipose tissue *in vitro*. Moreover, they reduced the release of pro-inflammatory mediators and expression of adhesion molecules in monocytes (77). Therefore, they could represent a promising tool in the treatment of obesity-related inflammation and insulin resistance.

## Author Contributions

GU and IB wrote the manuscript. CP and ET selected the articles. AM and DL realized the figures. EM revised the manuscript.

### Conflict of Interest

The authors declare that the research was conducted in the absence of any commercial or financial relationships that could be construed as a potential conflict of interest.
